# Baseline Neutrophil‐To‐Lymphocyte Ratio as a Risk Stratification Marker for Severe Checkpoint Inhibitor Pneumonitis in Patients With Non‐Small Cell Lung Cancer: A Two‐Cohort Study With Retrospective Discovery and Prospective Validation

**DOI:** 10.1002/resp.70267

**Published:** 2026-06-04

**Authors:** Tetsu Hirakawa, Kakuhiro Yamaguchi, Shun Takao, Nobuhiro Kanaji, Naoko Matsumoto, Kozue Miyazaki, Tamio Okimoto, Chihiro Ando, Hisao Higo, Masaaki Yanai, Koji Kurose, Yasunari Nagasaki, Masako Watanabe, Kosuke Oyama, Yuko Toyoda, Yoshihiro Taguchi, Shimpei Tada, Kiyofumi Shimoji, Shinjiro Sakamoto, Yasushi Horimasu, Takeshi Masuda, Taku Nakashima, Hiroshi Iwamoto, Hironobu Hamada, Noboru Hattori

**Affiliations:** ^1^ Department of Molecular and Internal Medicine Graduate School of Biomedical and Health Sciences, Hiroshima University Hiroshima Japan; ^2^ Department of Respiratory Medicine Hiroshima University Hospital Hiroshima Japan; ^3^ Department of Respiratory Internal Medicine Hiroshima City Hiroshima Citizens Hospital Hiroshima Japan; ^4^ Department of Internal Medicine, Division of Hematology, Rheumatology, and Respiratory Medicine Faculty of Medicine, Kagawa University Kagawa Japan; ^5^ Department of Respiratory Medicine Hiroshima Red Cross Hospital & Atomic‐Bomb Survivors Hospital Hiroshima Japan; ^6^ Department of Respiratory Medicine National Hospital Organization Higashihiroshima Medical Center Higashihiroshima Japan; ^7^ Department of Internal Medicine, Division of Medical Oncology & Respiratory Medicine Shimane University Faculty of Medicine Izumo Japan; ^8^ Department of Respiratory Medicine Japanese Red Cross Okayama Hospital Okayama Japan; ^9^ Department of Allergy and Respiratory Medicine Okayama University Hospital Okayama Japan; ^10^ Department of Respiratory Medicine and Rheumatology Tottori University Hospital Tottori Japan; ^11^ Department of Respiratory Medicine Kawasaki Medical School Okayama Japan; ^12^ Department of General Internal Medicine 4 Kawasaki Medical School Okayama Japan; ^13^ Department of Respiratory Medicine Hiroshima City North Medical Care Asa Citizens Hospital Hiroshima Japan; ^14^ Department of Respiratory Medicine and Allergology Kochi Medical School, Kochi University Kochi Japan; ^15^ Department of Internal Medicine Japanese Red Cross Kochi Hospital Kochi Japan; ^16^ Department of Cardiology, Pulmonology, Hypertension and Nephrology Ehime University Graduate School of Medicine Ehime Japan; ^17^ Department of Physical Analysis and Therapeutic Sciences, Graduate School of Biomedical and Health Sciences Hiroshima University Hiroshima Japan

**Keywords:** biomarker, checkpoint inhibitor pneumonitis, immune checkpoint inhibitor, neutrophil‐to‐lymphocyte ratio, non‐small‐cell lung cancer

## Abstract

**Background and Objective:**

Severe checkpoint inhibitor pneumonitis (CIP), particularly when it develops within 6–12 weeks, is a potentially fatal adverse event in patients with non‐small cell lung cancer (NSCLC) receiving immune checkpoint inhibitors (ICIs). Although neutrophil‐to‐lymphocyte ratio (NLR) has been reported to predict immune‐related adverse events, its role in severe CIP remains unclear.

**Methods:**

The discovery cohort retrospectively enrolled 102 patients with NSCLC treated with ICI monotherapy at Hiroshima University Hospital. The validation cohort prospectively enrolled 191 NSCLC patients receiving first‐line treatment, including ICI, at 15 institutions. Severe CIP was defined as grade 3–5 pneumonitis occurring within 3 months of treatment initiation. The cut‐off level of the baseline NLR was identified by receiver operating characteristic curve analysis in the discovery cohort, and the predictive accuracy was analyzed and confirmed in both cohorts.

**Results:**

Severe CIP was observed in seven (6.9%) and 11 (5.8%) patients in the discovery and validation cohorts, respectively. In the discovery cohort, the baseline NLR was higher in patients with severe CIP than in those without (17.29 [4.76–20.73] vs. 3.70 [2.64–6.68], *p* = 0.006), and the cut‐off level of the baseline NLR was set at 4.75 (AUC 0.81; sensitivity 85.7%, specificity 64.2%). The incidence of severe CIP was significantly higher in patients with higher baseline NLR levels (≥ 4.75) than in those without, in both cohorts (discovery: 15.0% vs. 1.6%, *p* = 0.009; validation: 10.3% vs. 3.3%, *p* = 0.046).

**Conclusion:**

Baseline NLR is a promising risk stratification marker for severe CIP.

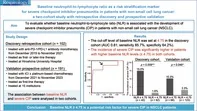

## Graphical abstract

The association between baseline neutrophil‐to‐lymphocyte ratio (NLR) and severe checkpoint inhibitor pneumonitis (CIP) was analyzed in discovery retrospective and validation prospective cohorts. Higher baseline NLR (≥ 4.75) was associated with increased severe CIP incidence in both cohorts. Baseline NLR is a promising risk stratification marker for severe CIP.

## 1 Introduction

Immune checkpoint inhibitors (ICIs) significantly improve the prognosis of patients with non‐small‐cell lung cancer (NSCLC) [1]. While ICIs can cause immune‐related adverse events (irAEs) such as thyroid dysfunction, skin rash, diarrhoea, and colitis, which are not typically observed with conventional chemotherapy, the development of these irAEs has generally been associated with a favourable prognosis [2, 3]. In contrast, checkpoint inhibitor pneumonitis (CIP) has been reported to occur more frequently in patients with NSCLC than in those with other carcinomas, and patients who develop CIP tend to have a worse prognosis [4, 5]. In particular, severe CIP, which is typically graded 3–5 and develops within 6–12 weeks, is associated with a poorer prognosis [6, 7, 8, 9]. These findings underscore the necessity of overcoming severe CIP by developing a risk assessment biomarker, early diagnosis method, and appropriate treatment.

Several risk factors have been proposed for the risk assessment of CIP, including a history of interstitial lung disease (ILD) or interstitial lung abnormalities, prior thoracic radiation therapy, and use of anti‐programmed cell death 1 (PD‐1) antibody [10, 11, 12]. However, these factors alone do not allow for the reliable prediction of CIP development, and their reproducibility has not been confirmed prospectively. Additionally, the association between these risk factors and CIP severity has not been elucidated. Therefore, the identification of novel and more robust risk assessment biomarkers for severe CIP that can be used in daily clinical practice is warranted.

The neutrophil‐to‐lymphocyte ratio (NLR), calculated as the absolute neutrophil count (ANC) divided by the absolute lymphocyte count (ALC), is an inexpensive, objective, and widely available biomarker [13]. The NLR is recognized as an indicator of systemic inflammatory response and immune status, and a high baseline NLR has been reported to be a poor prognostic indicator in several malignancies treated with immunotherapy [14]. Concerning the association between NLR and irAEs, paradoxical reports have been reported depending on the severity and type of irAEs. For example, although several studies have reported an association between a low NLR and an increased incidence of all‐grade irAEs [15, 16], other studies have demonstrated that a high NLR is associated with severe grade irAEs [17, 18]. When focusing on CIP, NLR was elevated at the onset of CIP compared to baseline and was correlated with disease severity [19, 20]. These findings suggest that a high baseline NLR may serve as a risk assessment marker for CIP, particularly for severe diseases. Nevertheless, the relationship between baseline NLR and severe CIP remains unclear.

Therefore, this study aimed to evaluate the association between baseline NLR and severe CIP in patients with NSCLC through both a retrospective discovery cohort and a prospective validation cohort.

## 2 Methods

### 2.1 Study Population and Design

The discovery cohort retrospectively included 119 patients with unresectable advanced or recurrent NSCLC who were treated with anti‐PD‐1/programmed cell death ligand 1 (PD‐L1) antibody monotherapy (nivolumab, pembrolizumab, or atezolizumab) as first‐ or later‐line treatment at Hiroshima University Hospital from December 2015 to November 2021 (Figure S1). Seventeen patients who did not provide written consent for the biomarker research were excluded. Finally, 102 patients were included in the discovery cohort.

The validation cohort prospectively included 200 patients with unresectable advanced or recurrent NSCLC without pre‐existing ILD who were treated with anti‐PD‐1/PD‐L1 antibody (nivolumab, pembrolizumab, atezolizumab, or durvalumab) or in combination with anti‐cytotoxic T‐lymphocyte antigen‐4 (CTLA‐4) antibody (ipilimumab or tremelimumab) and/or platinum‐based chemotherapy as first‐line treatment at 15 institutions from December 2021 to November 2023 (a multicentre prospective observational study to identify the risk of CIP; CS‐Lung004 study) (Figure S1). Before enrollment, patients with metastatic or recurrent NSCLC and those with locally advanced NSCLC were screened. Based on a multiplex assay for detecting driver oncogene mutations, such as Oncomine Dx and Amoy Dx, this study excluded patients with NSCLC harbouring driver oncogene mutations who were to be treated with molecular target therapy, such as epidermal growth factor receptor‐tyrosine kinase inhibitors, in the first‐line treatment. If driver oncogenes could not be investigated owing to inadequate tumour samples, the patients were enrolled. This study also excluded patients who had preexisting ILD, those who were treated with chemotherapy for lung cancer within 6 months, those who were irradiated radically within 1 year, those who were treated with systemic chemotherapy for other cancers within 6 months, and those with a history of ICI treatment. Nine patients who discontinued treatment within 3 months for reasons other than disease progression or adverse events were excluded. Finally, 191 patients were included in the validation cohort.

First, in the discovery cohort, the baseline NLR cut‐off for assessing the risk of severe CIP, defined as grade 3–5 occurring within 3 months, was identified using receiver operating characteristic (ROC) curve analysis. Subsequently, this study analyzed the predictive accuracy of the NLR cut‐off level for severe CIP in both the discovery and validation cohorts. Finally, a pooled analysis was conducted by integrating the discovery and validation cohorts to enhance the statistical power and robustness of the study, resulting in a combined cohort of 293 patients with unresectable advanced or recurrent NSCLC who were treated with ICIs.

This study was approved by the Ethics Committee of Hiroshima University Hospital (approval numbers: E2004–0326 and E2021–2656) and was conducted in accordance with the ethical standards established in the Helsinki Declaration. Each institution participating in the CS‐Lung004 study approved the study protocol. All the participants provided written informed consent for the use of their medical records.

### 2.2 Diagnosis of CIP in the Retrospective Discovery Cohort and the Prospective Validation Cohort

In the retrospective discovery cohort, CIP was diagnosed by two experienced pulmonologists, including the attending physician, based on the following criteria: (1) new ground‐glass abnormality or bilateral consolidation on chest radiography or computed tomography (CT); (2) no indication of apparent heart failure, pulmonary invasion of lung cancer, or pulmonary infection (no improvement with antibiotics when administered and negative sputum and/or blood cultures when obtained); and (3) lung injury developed within 6 weeks after the last administration of ICI therapy. Tumour progression, including lymphangitic carcinomatosis, was assessed and excluded based on CT findings and clinical evaluation. Radiation‐related lung injury was excluded based on the irradiated field and the distribution of radiographic abnormalities. Lung injuries occurring after the initiation of subsequent therapies were not classified as CIP. In this study, CIP that developed within 3 months after the initiation of ICI treatment was counted, and these were classified as mild CIP (grades 1–2) and severe CIP (grades 3–5) according to the Common Terminology Criteria for Adverse Events version 5.0 [9].

In the prospective validation cohort, CIP was prospectively diagnosed based on the criteria as described above. The follow‐up schedule was as follows: before treatment, chest radiography and CT were performed to confirm the absence of ILD. To detect CIP, chest radiographs, oxygenation evaluated using SpO_2_, and clinical symptoms were checked at every ICI administration visit, according to the standard schedule of each regimen. If any abnormalities were detected on chest radiographs, or in oxygenation levels or in clinical symptoms, a chest CT was performed. Even if no abnormalities were noted, chest CT was performed 3 months after treatment initiation to confirm the absence of CIP. The final diagnoses of CIP and CIP grade were prospectively judged and confirmed by the attending pulmonologist and another experienced pulmonologist.

### 2.3 Peripheral Blood Data Collection

In the discovery cohort, baseline peripheral blood data were obtained from routine laboratory tests performed within 7 days before the first administration of anti‐PD‐1/PD‐L1 therapy, regardless of treatment line. When more than one test was available within the window, we used the sample closest to anti‐PD‐1/PD‐L1 antibody therapy initiation.

In the validation cohort (CS‐Lung004 study), blood sampling for serum/plasma banking was prospectively scheduled to be performed concurrently with routine pre‐treatment blood draws in daily clinical practice and was required to be performed within 14 days before treatment initiation. Baseline peripheral blood data obtained at the same timepoint were used in this study.

In both cohorts, peripheral blood data were also obtained at the time of CIP diagnosis.

### 2.4 Statistical Analysis

Continuous variables are presented as median [interquartile range]. Differences among groups were evaluated using Pearson's chi‐squared and Mann–Whitney U tests. The cut‐off level of the baseline NLR to predict severe CIP was identified by ROC analysis in the discovery cohort, and the predictive accuracy was analyzed and confirmed by Pearson's chi‐squared test in the discovery and validation cohorts. For ROC analyses, the area under the curve (AUC) and its 95% confidence interval (CI) were calculated. Univariate and multivariate logistic regression analyses were conducted to identify independent risk factors for severe CIP and to estimate the odds ratios and 95% CIs. Statistical significance was set at *p* < 0.05. All data analyses were performed using JMP version 18.2.1 (SAS Institute Inc., Cary, NC, USA).

## 3 Results

### 3.1 Patient Characteristics

The baseline patient characteristics are shown in Table 1. In the discovery cohort, 69 patients (67.6%) received ICI as second‐line or later‐line treatment. Anti‐PD‐1 antibody and anti‐PD‐L1 antibody were administered to 85 patients (83.3%) and 17 patients (16.7%), respectively. In the validation cohort, all patients received ICI as first‐line treatment. Anti‐PD‐1 antibody and anti‐PD‐L1 antibody were administered to 165 patients (85.3%) and 28 patients (14.7%), respectively. Anti‐CTLA‐4 antibody was combined with anti‐PD‐1/PD‐L1 antibody therapy in 41 patients (21.5%) and chemotherapy was combined in 130 patients (68.1%).

**TABLE 1:** Baseline characteristics.

	Discovery cohort			Validation cohort		
	Severe CIP (+) (*n* = 7)	Severe CIP (−) (*n* = 95)	*p*‐value	Severe CIP (+) (*n* = 11)	Severe CIP (−) (*n* = 180)	*p*‐value
Age (years)	68 (64–74)	72 (65–77)	0.423	69 (66–75)	73 (64–77)	0.603
Sex, male, *n* (%)	6 (85.7)	65 (68.4)	0.337	11 (100.0)	148 (82.2)	0.125
Smoking history, current or former, *n* (%)	7 (100.0)	77 (81.1)	0.204	11 (100.0)	159 (88.3)	0.230
PS, ≥ 2, *n* (%)	1 (14.3)	21 (22.1)	0.627	0 (0.0)	16 (8.9)	0.302
Interstitial lung disease, +, *n* (%)	2 (28.6)	16 (16.8)	0.432	0 (0.0)	0 (0.0)	—
COPD, +, *n* (%)	2 (28.6)	31 (32.6)	0.825	4 (36.4)	61 (33.9)	0.867
Previous thoracic RT, +, *n* (%)	2 (28.6)	32 (33.7)	0.782	2 (18.2)	6 (3.3)	0.017*
Stage, *n* (%)			0.551			0.047*
III	0 (0.0)	8 (8.4)		0 (0.0)	15 (8.3)	
IV	4 (57.1)	61 (64.2)		7 (63.6)	144 (80.0)	
Recurrence	3 (42.9)	26 (27.4)		4 (36.4)	21 (11.7)	
Histological type, *n* (%)			0.220			0.559
Squamous	0 (0.0)	17 (17.9)		2 (18.2)	47 (26.1)	
Non‐Squamous	7 (100.0)	78 (82.1)		9 (81.8)	133 (73.9)	
Driver mutation, +, *n* (%)	1 (14.3)	18 (18.9)	0.760	0 (0.0)	13 (7.2)	0.356
PD‐L1 TPS, *n* (%)			0.835			0.865
≥ 50%	2 (28.6)	40 (42.1)		5 (45.5)	82 (45.6)	
1%–49%	2 (28.6)	18 (18.9)		3 (27.3)	57 (31.7)	
< 1%	1 (14.3)	8 (8.4)		2 (18.2)	34 (18.9)	
Unknown	2 (28.6)	29 (30.5)		1 (9.1)	7 (3.9)	
ICI treatment line, *n* (%)			0.058			—
1st	0 (0.0)	33 (34.7)		11 (100.0)	180 (100.0)	
2nd or later	7 (100.0)	62 (65.3)		0 (0.0)	0 (0.0)	
Treatment regimen, *n* (%)						
Anti‐PD‐1 antibody	6 (85.7)	79 (83.2)	0.861	11 (100.0)	152 (84.4)	0.157
Anti‐PD‐L1 antibody	1 (14.3)	16 (16.8)	0.861	0 (0.0)	28 (15.6)	0.157
With anti‐CTLA‐4 antibody	0 (0.0)	0 (0.0)	—	3 (27.3)	38 (21.1)	0.629
With chemotherapy	0 (0.0)	0 (0.0)	—	8 (72.7)	122 (67.8)	0.733
Blood test results						
WBC (/μL)	9690 (6100–17,990)	6690 (4950–8660)	0.080	7400 (5610–12,470)	7495 (6208–8998)	0.526
ANC (/μL)	8207 (3750–13,536)	4563 (3367–9836)	0.044*	5927 (4011–9379)	5124 (4168–6535)	0.240
ALC (/μL)	788 (475–1507)	1185 (869–1650)	0.087	1128 (807–1331)	1457 (1004–1833)	0.155
NLR	17.29 (4.76–20.73)	3.70 (2.64–6.68)	0.006**	5.55 (3.69–7.35)	3.67 (2.72–5.70)	0.021*
LDH (U/L)	235 (191–539)	204 (171–276)	0.314	199 (167–271)	208 (175–303)	0.601
Alb (g/dL)	3.1 (2.3–3.8)	3.5 (3.1–3.9)	0.162	3.3 (3.0–3.6)	3.7 (3.3–4.0)	0.020*
CRP (mg/dL)	1.0 (0.3–13.8)	1.2 (0.2–4.5)	0.275	4.0 (0.4–4.2)	0.7 (0.2–2.7)	0.075

*Note:* Data are presented as median (interquartile range) unless stated otherwise. *p < 0.05 and **p < 0.01, comparison between severe CIP (+) and severe CIP (−) using the Mann–Whitney U test or Pearson's chi‐square test.

Abbreviations: Alb, albumin; ALC, absolute lymphocyte count; ANC, absolute neutrophil count; COPD, chronic obstructive pulmonary disease; CRP, C‐reactive protein; ICI, immune checkpoint inhibitor; LDH, lactate dehydrogenase; NLR, neutrophil‐to‐lymphocyte ratio; PD‐1, programmed cell death 1; PD‐L1, programmed cell death ligand 1; PS, performance status; RT, radiotherapy; TPS, tumour proportion score; WBC, white blood cell.

### 3.2 Predictive Potential of Baseline NLR for Severe CIP in the Discovery Cohort

**FIGURE 1:**
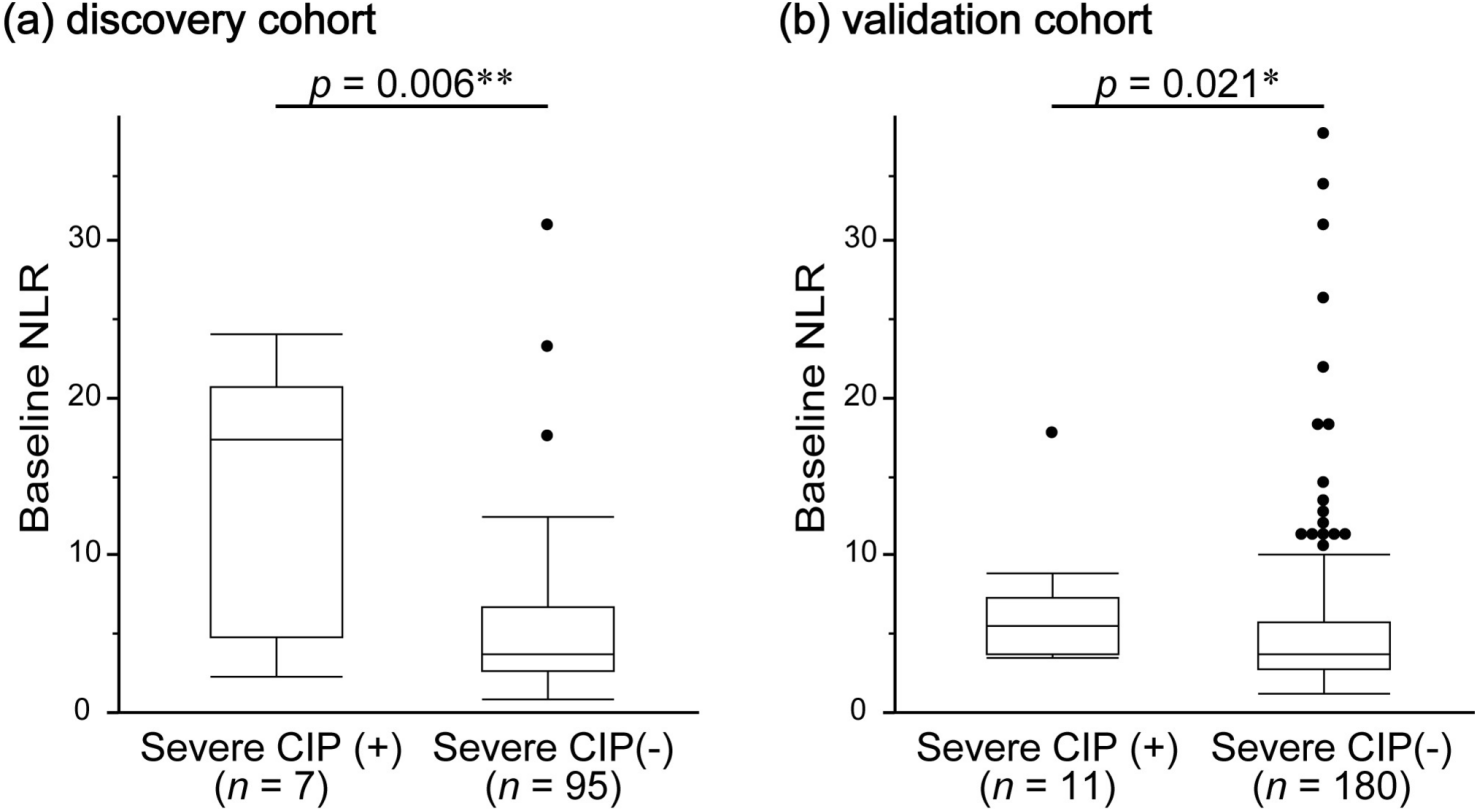
Baseline neutrophil‐to‐lymphocyte ratio (NLR) in patients with and without severe checkpoint inhibitor pneumonitis (CIP) Baseline NLR was significantly higher in patients who developed severe CIP compared to those who did not in each cohort: (a) in the discovery cohort, 17.29 [4.76–20.73] vs. 3.70 [2.64–6.68], *p* = 0.006; (b) in the validation cohort, 5.55 [3.69–7.35] vs. 3.67 [2.72–5.70], *p* = 0.021. Boxes represent the interquartile range (25th–75th percentiles), solid lines within the boxes indicate the medians, and whiskers denote the 10th and 90th percentiles. Outliers were plotted as individual points. *p < 0.05 and **p < 0.01, Mann–Whitney U test. CIP, checkpoint inhibitor pneumonitis; NLR, neutrophil‐to‐lymphocyte ratio.

**FIGURE 2:**
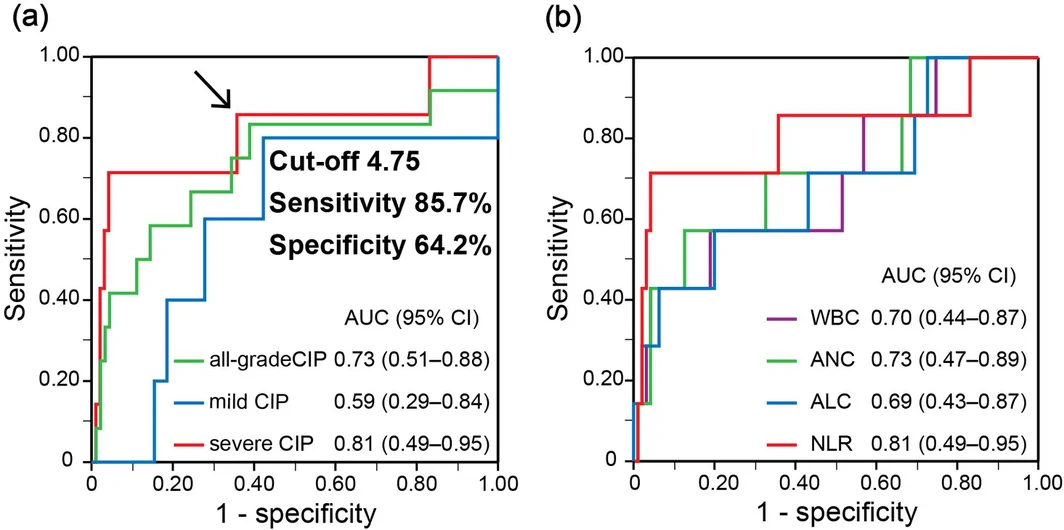
Receiver operating characteristic (ROC) curves analyses to identify a cut‐off level of neutrophil‐to‐lymphocyte ratio (NLR). (a) In the discovery cohort, receiver operating characteristic (ROC) curve analysis showed that the area under the curve (AUC) of baseline NLR for predicting all‐grade checkpoint inhibitor pneumonitis (CIP), mild CIP, and severe CIP were 0.73 (95% confidence interval [CI]: 0.51–0.88), 0.59 (95% CI: 0.29–0.84), and 0.81 (95% CI: 0.49–0.95), respectively. The baseline NLR cut‐off level for predicting severe CIP was set at 4.75 (sensitivity = 85.7% and specificity = 64.2%). (b) The predictive performance of each baseline biomarker for severe CIP was evaluated by ROC curve analysis, and the NLR showed the highest AUC for severe CIP compared to white blood cell count (WBC), absolute neutrophil count (ANC), and absolute lymphocyte count (ALC). ALC, absolute lymphocyte count; ANC, absolute neutrophil count; AUC, area under the curve; CI, confidence interval; CIP, checkpoint inhibitor pneumonitis; NLR, neutrophil‐to‐lymphocyte ratio; ROC, receiver operating characteristic; WBC, white blood cell count.

**FIGURE 3:**
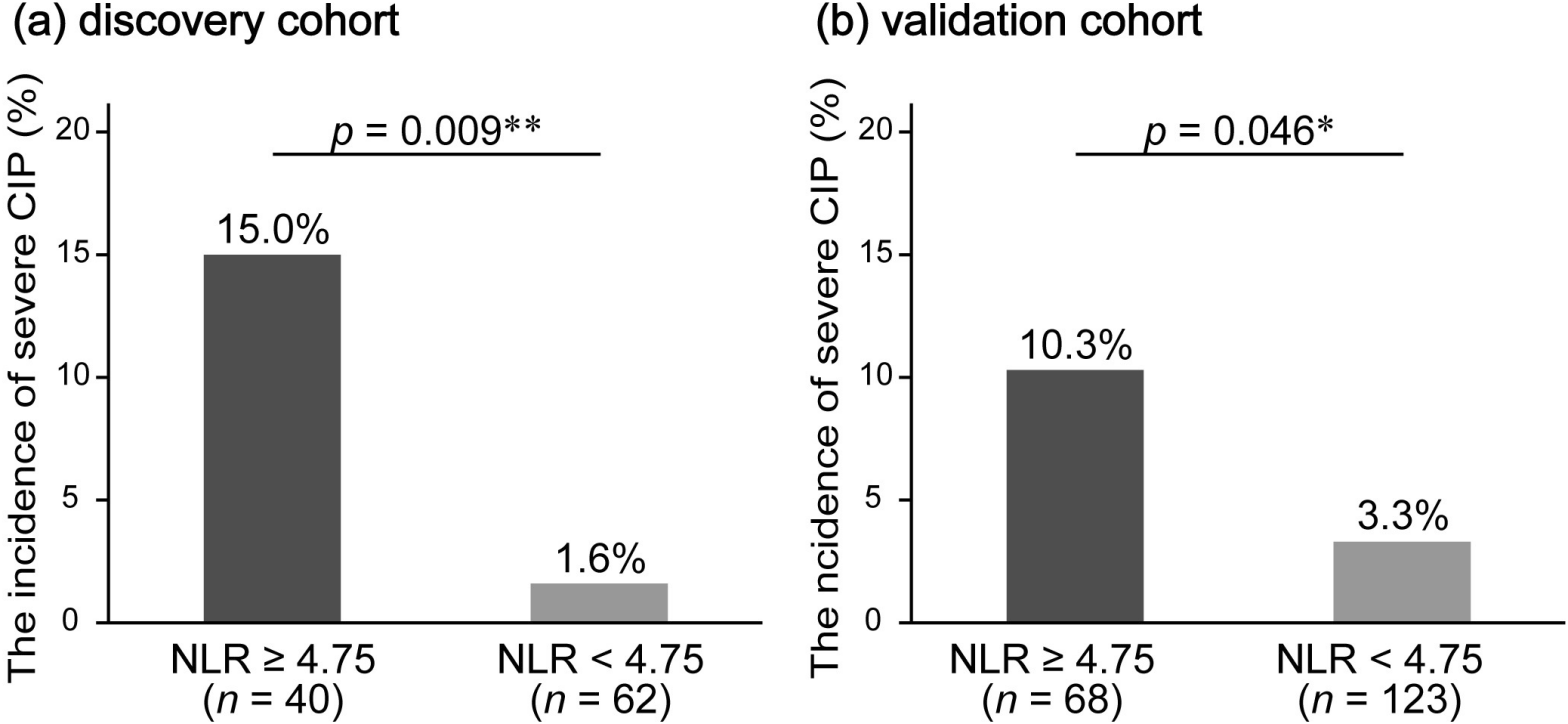
Comparison of the incidence of severe checkpoint inhibitor pneumonitis (CIP) stratified by baseline neutrophil‐to‐lymphocyte ratio (NLR) cut‐off level. The incidence of severe CIP was significantly higher in patients with higher baseline NLR (≥ 4.75) than in those with lower baseline NLR (< 4.75) in each cohort: (a) discovery cohort, 15.0% vs. 1.6%, *p* = 0.009; (b) validation cohort, 10.3% vs. 3.3%, *p* = 0.046. *p < 0.05 and **p < 0.01, Pearson's chi‐square test. CIP, checkpoint inhibitor pneumonitis; NLR, neutrophil‐to‐lymphocyte ratio.

In the discovery cohort, CIP was observed in 12 patients (11.8%), of whom seven (6.9%) developed severe CIP. Baseline NLR was significantly higher in the patients with severe CIP than in those without severe CIP (17.29 [4.76–20.73] vs. 3.70 [2.64–6.68], *p* = 0.006) (Figure 1a), whereas no significant difference was observed between the patients with mild CIP and those without CIP (6.35 [2.59–9.25] vs. 3.63 [2.62–6.28], *p* = 0.359) (Figure S2a). ROC curve analysis showed that the AUC of the baseline NLR for predicting all‐grade CIP, mild CIP, and severe CIP were 0.73 (95% CI: 0.51–0.88), 0.59 (95% CI: 0.29–0.84), and 0.81 (95% CI: 0.49–0.95), respectively (Figure 2a). The highest AUC was observed for severe CIP, for which the baseline NLR cut‐off level was set at 4.75 (sensitivity = 85.7% and specificity = 64.2%), taking into consideration previous studies reporting NLR cut‐off levels typically ranging from 3 to 5 [15, 16, 21]. The baseline characteristics according to baseline NLR levels are shown in Table S1. When patients were stratified by this cut‐off baseline NLR level, the incidence of severe CIP was significantly higher in patients with a higher baseline NLR (≥ 4.75) than in those with a lower baseline NLR (< 4.75) (15.0% vs. 1.6%, *p* = 0.009) (Figure 3a). In contrast, the incidence of mild CIP did not differ significantly between patients with a higher baseline NLR (≥ 4.75) and those with a lower baseline NLR (< 4.75) (8.9% vs. 3.3%, *p* = 0.246) (Figure S2c).

### 3.3 Confirmation of Baseline NLR Utility to Predict Severe CIP in the Validation Cohort

In the validation cohort, CIP was observed in 20 patients (10.5%), of whom 11 (5.8%) developed severe CIP. The baseline NLR was significantly higher in patients with severe CIP than in those without severe CIP (5.55 [3.69–7.35] vs. 3.67 [2.72–5.70], *p* = 0.021) (Figure 1b), whereas no significant difference was observed between patients with mild CIP and those without CIP (3.92 [2.54–5.49] vs. 3.67 [2.73–5.70], *p* = 0.653) (Figure S2b). When patients were stratified by the cut‐off baseline NLR set in the discovery cohort, the incidence of severe CIP was significantly higher in patients with a higher baseline NLR (≥ 4.75) than in those with a lower baseline NLR (< 4.75) (10.3% vs. 3.3%, *p* = 0.046) (Figure 3b). In contrast, the incidence of mild CIP did not differ significantly between patients with a higher baseline NLR (≥ 4.75) and those with a lower baseline NLR (< 4.75) (4.9% vs. 5.0%, *p* = 0.971) (Figure S2d).

### 3.4 Pooled Analysis of the Association Between Baseline NLR and Severe CIP

We conducted a pooled analysis by integrating the discovery and validation cohorts, resulting in 293 patients with NSCLC treated with ICIs. In the pooled cohort, baseline NLR was significantly higher in patients with severe CIP than in those without severe CIP (6.16 [4.15–17.41] vs. 3.65 [2.69–5.90], *p* < 0.001) (Figure S3a). Moreover, the incidence of severe CIP was significantly higher in patients with a higher baseline NLR (≥ 4.75) than in those with a lower baseline NLR (< 4.75) (12.0% vs. 2.7%, *p* = 0.001) (Figure S3b). The subgroup analyses stratified by treatment regimen showed that the incidence of severe CIP tended to be higher in patients with a higher baseline NLR (≥ 4.75) than in those with a lower baseline NLR (< 4.75) in regimens containing anti‐PD‐1 antibodies, such as pembrolizumab and nivolumab, but not in those containing anti‐PD‐L1 antibodies (Figure S4).

Subsequently, logistic regression analyses were performed to identify the independent risk factors for severe CIP. In the pooled cohort, univariate and multivariate analyses demonstrated that a baseline NLR ≥ 4.75 was significantly associated with a higher incidence of severe CIP (Table 2). Comparable trends were observed in the individual discovery and validation cohorts (Table S2).

**TABLE 2:** Logistic regression analyses for identifying the risk of severe checkpoint inhibitor pneumonitis (CIP) in the pooled cohort.

Variables	Pooled cohort (*n* = 293)		
	OR	95% CI	*p*‐value
Univariate analysis			
Age, years	0.996	0.952–1.048	0.858
Sex, male	4.948	0.985–89.990	0.053
Smoking history, Current or Former	Not available^a^	Not available^a^	0.021*
PS, ≥ 2	0.378	0.021–1.930	0.286
Interstitial lung disease, +	2.023	0.304–7.988	0.409
History of COPD, +	0.995	0.337–2.648	0.992
Previous thoracic RT, +	1.758	0.486–5.278	0.353
Stage, IV or recurrence	Not available^a^	Not available^a^	0.081
Histological type, squamous	0.412	0.064–1.500	0.198
Driver mutation, positive	0.463	0.025–2.380	0.413
PD‐L1 TPS, ≥ 50%	0.798	0.286–2.088	0.649
ICI treatment line, 1st	0.457	0.173–1.289	0.134
ICI treatment, anti‐PD‐1 antibody	3.238	0.639–59.058	0.182
ICI treatment, with anti‐CTLA‐4 antibody	1.247	0.280–4.005	0.742
ICI treatment, with chemotherapy	1.003	0.373–2.620	0.995
NLR, ≥ 4.75	4.926	1.799–15.730	0.002**
Alb, ≥ 3.5 g/dL	0.436	0.162–1.141	0.090
CRP, ≥ 1.0 mg/dL	1.500	0.575–4.039	0.406
Multivariate analysis			
Smoking history, current or former	Not available^a^	Not available^a^	0.012*
NLR, ≥ 4.75	5.369	1.949–17.226	0.001**

*Note:* *p < 0.05 and **p < 0.01, logistic regression analysis.

Abbreviations: Alb, albumin; CI, confidence interval; CIP, checkpoint inhibitor pneumonitis; COPD, chronic obstructive pulmonary disease; CRP, C‐reactive protein; CTLA4, cytotoxic T‐lymphocyte antigen 4; ICI, immune checkpoint inhibitor; OR, odds ratio; PD‐1, programmed cell death‐1; PD‐L1, programmed cell death ligand‐1; PS, performance status; TPS, tumour proportion score.

^a^ OR (odds ratio) and 95% confidence interval (CI) could not be calculated because only one group developed severe CIP.

### 3.5 Comparison of the Predictive Performance of Biomarkers Derived From Complete Blood Count for Severe CIP

White blood cell count (WBC), ANC, and ALC are the constituent components of the NLR and are used in the calculation. To evaluate which of these markers had superior predictive ability for the development of severe CIP, we additionally calculated and compared the AUC for each baseline parameter in the discovery cohort (Figure 2b). Among these biomarkers, NLR demonstrated the highest AUC, which was consistent with the analyses of the validation and pooled cohorts (Figure S5).

### 3.6 Association Between NLR at the Time of CIP Diagnosis and CIP Severity

Among patients who developed CIP in the pooled cohort, the median NLR at the time of CIP diagnosis was 5.92 (2.55–10.04). Both baseline NLR and NLR at the time of CIP diagnosis were significantly higher in patients with severe CIP than in those with mild CIP (6.16 [4.15–17.41] vs. 4.15 [2.62–6.11], *p* = 0.040, and 7.28 [4.28–12.0] vs. 3.75 [1.82–6.83], *p* = 0.023, respectively) (Figure S6a,b). In contrast, the absolute change between baseline NLR and NLR at the time of CIP diagnosis was not significantly associated with CIP severity (Figure S6c).

## 4 Discussion

This study investigated the association between the baseline NLR and the incidence of severe CIP in patients with NSCLC in a retrospective discovery cohort and a prospective validation cohort. The cut‐off level of baseline NLR was set at 4.75 in the discovery cohort. In both cohorts, a high baseline NLR (≥ 4.75) was consistently and significantly associated with a high incidence of severe CIP. In addition, a comparison of the predictive performance of blood biomarkers revealed that NLR had the highest AUC among WBC, ANC, and ALC. These data suggest that baseline NLR may serve as a promising risk stratification marker for severe CIP, which can be used in daily clinical practice.

This was the first study to elucidate the association between a high baseline NLR and the risk of severe CIP. Previous studies have demonstrated that elevated NLR is associated with the incidence of various severe acute lung injuries, including acute exacerbation of idiopathic pulmonary fibrosis, acute respiratory distress syndrome, COVID‐19‐related pneumonia, and radiation pneumonitis [22, 23, 24, 25]. These findings suggest that an elevated NLR may reflect a systemic inflammatory state that contributes to the development of serious pulmonary damage.

Inconsistent with this study, high NLR at baseline has not been identified as a risk factor for CIP in previous studies, although several reports have shown that NLR increases at CIP onset from baseline and is higher in severe CIP than in mild disease at CIP diagnosis [19, 26]. This discrepancy is likely attributable to previous studies assessing the association between baseline NLR and the risk of CIP without specifically accounting for disease severity [27, 28, 29]. Our analysis revealed that the baseline NLR was significantly higher in patients with severe CIP than in those without, whereas no such association was observed when analyzed by focusing on cases of mild CIP. Additionally, the AUC of 0.81 for predicting severe CIP using NLR was higher than that of 0.59 for predicting mild CIP. Consistent with our data, several reports of irAEs have shown that the baseline NLR is higher in severe cases than in mild cases [17, 18]. These findings indicate that NLR may serve as a more specific risk stratification marker of life‐threatening forms of CIP rather than milder forms. Identifying patients with a high baseline NLR may help clinicians stratify risk and implement closer monitoring strategies for early detection and management of severe CIP.

For the prediction of severe CIP, NLR, which combines two parameters into a ratio, demonstrated better predictive accuracy than individual parameters such as WBC, ANC, or ALC. As shown in Table 1, the patients who developed severe CIP tended to have higher ANC and lower ALC; therefore, their ratio (NLR), which is calculated as the ANC divided by the ALC, might capture this imbalance more robustly, thereby showing the highest predictive utility for severe CIP.

Although the mechanistic association between severe CIP and a high NLR is unclear, this study demonstrated that a high baseline NLR, indicative of increased neutrophil levels, was significantly associated with the development of severe CIP. Neutrophils extravasate from pulmonary capillary endothelial cells into the alveolar interstitium through interactions with adhesion molecules such as selectin, integrin, intracellular adhesion molecule‐1, and vascular cell adhesion molecule‐1 [30, 31]. Next, activated neutrophils form neutrophil extracellular traps and release histones, along with enzymes such as proteinases, transforming growth factor‐beta, and reactive oxygen species, which amplify the inflammatory response and contribute to lung injury [31, 32]. Additionally, neutrophils may further exacerbate inflammation by releasing damage‐associated molecular patterns, including high‐mobility group box 1 (HMGB1) and S100 proteins [32, 33]. Notably, our recent study demonstrated that elevated serum HMGB1 level serves as a predictive biomarker for severe CIP in NSCLC patients [34]. These data indicate one potential mechanism to develop severe CIP: that excessive activation and accumulation of neutrophils may be critical contributors to the development and progression of CIP.

Another possibility is that T‐cell dysregulation, characterized by an increase in T helper 17 (Th17) cells and a reduction in regulatory T (Treg) cells, contributes to the pathophysiology of severe CIP [2, 35, 36]. Th17 cells mainly produce interleukin‐17, which accelerates the migration of neutrophils and regulates immune homeostasis related to the pathogenesis of various autoimmune diseases and pulmonary fibrosis [37, 38]. Additionally, Tregs suppress inflammation and maintain immune homeostasis; therefore, their reduction or dysfunction can exacerbate lung injury [39]. Notably, the Th17/Treg ratio was positively correlated with NLR in several diseases [40, 41]. Moreover, the elevated Th17/Treg ratio at CIP onset was significantly higher than that at baseline [36]. Although hypothetical, these findings suggest that the development of CIP involves a complex interplay between T cell dysregulation and increased inflammatory cytokines, and therefore a high NLR may serve as a promising risk stratification marker of severe CIP by reflecting these immunological changes.

This study has several limitations. First, the number of cases of severe CIP was small, so it is possible that the number of cases was insufficient to perform ROC analysis and determine cutoff values. This is unavoidable given the prospective case collection and incidence rate of CIP. Second, although CIP was diagnosed by two experienced pulmonologists, the imaging findings for CIP diagnosis were not centrally reviewed. Therefore, some interobserver variability in the radiographic assessment and differential diagnosis cannot be completely excluded. Third, although irAEs are commonly ascribed to lymphocyte activation and bronchoalveolar lavage fluid from patients with CIP often shows lymphocytosis, our findings appear counterintuitive. Notably, lymphopenia has been reported in severe CIP and other high‐grade irAEs, suggesting that severe irAEs may follow a pathophysiology distinct from that of simple lymphocyte activation. To address this gap, further mechanistic studies are required to elucidate the processes underlying severe CIP. Fourth, because the predictive ability of NLR alone for severe CIP is limited, future studies should aim to improve the prediction accuracy by combining NLR with other promising biomarkers and by constructing multifactorial models that incorporate various inflammatory and immune‐related markers. Fifth, NLR was not collected longitudinally during treatment or systematically after CIP onset. Therefore, we were unable to assess whether dynamic changes in NLR during anti‐PD‐1/PD‐L1 therapy or changes in NLR after treatment for CIP were associated with the development or clinical course of CIP.

In conclusion, this study confirmed that a high baseline NLR (≥ 4.75) is significantly associated with an increased risk of developing severe CIP in patients with NSCLC. This association was consistently observed in the two independent cohorts including the multicentre prospective validation cohort. These findings suggest that the baseline NLR may serve as a readily available and promising risk stratification marker for identifying patients at high risk of severe CIP; however, further external validation in larger cohorts is needed.

## Author Contributions

**Tetsu Hirakawa:** conceptualization, data curation, formal analysis, investigation, resources, project administration, visualization, writing – original draft. **Kakuhiro Yamaguchi:** conceptualization, data curation, formal analysis, investigation, methodology, project administration, resources, validation, visualization, writing – original draft, writing – review and editing. **Shun Takao:** resources, writing – review and editing. **Nobuhiro Kanaji:** resources, writing – review and editing. **Naoko Matsumoto:** resources, writing – review and editing. **Kozue Miyazaki:** resources, writing – review and editing. **Tamio Okimoto:** resources, writing – review and editing. **Chihiro Ando:** resources, writing – review and editing. **Hisao Higo:** resources, writing – review and editing. **Masaaki Yanai:** resources, writing – review and editing. **Koji Kurose:** resources, writing – review and editing. **Yasunari Nagasaki:** resources, writing – review and editing. **Masako Watanabe:** resources, writing – review and editing. **Kosuke Oyama:** resources, writing – review and editing. **Yuko Toyoda:** resources, writing – review and editing. **Yoshihiro Taguchi:** resources, writing – review and editing. **Shimpei Tada:** resources, project administration, writing – review and editing. **Kiyofumi Shimoji:** resources, writing – review and editing. **Shinjiro Sakamoto:** resources, writing – review and editing. **Yasushi Horimasu:** resources, writing – review and editing. **Takeshi Masuda:** resources, writing – review and editing. **Taku Nakashima:** resources, writing – review and editing. **Hiroshi Iwamoto:** resources, supervision, writing – review and editing. **Hironobu Hamada:** resources, supervision, writing – review and editing. **Noboru Hattori:** resources, funding acquisition, supervision, writing – review and editing.

## Funding

This study was funded by The AMED Translational Research Grant Seeds A (2021) and Shino‐Test Corporation.

## Ethics Statement

This study was approved by the Ethics Committee of Hiroshima University Hospital, and all participants provided written informed consent for the use of medical records (Hiroshima University Hospital [E2004–0326 and E2021‐2656]).

## Conflicts of Interest

Kakuhiro Yamaguchi reports personal fees from Ono Pharmaceutical Co. Ltd., Chugai Pharmaceutical Co. Ltd., and Bristol Myers Squibb, and also reports grants from the AMED Translational Research Grant Seeds A (2021) and Shino‐Test Corporation. Masaaki Yanai reports personal fees from MSD K.K., Ono Pharmaceutical Co. Ltd., Chugai Pharmaceutical Co. Ltd., and AstraZeneca K.K. Takeshi Masuda reports personal fees from Daiichi‐Sankyo Co. Ltd., Taiho Pharmaceutical Co. Ltd., Nippon Boehringer Ingelheim Co. Ltd., Kyowa Kirin Co. Ltd., Eli Lilly Japan K.K., Ono Pharmaceutical Co. Ltd., Otsuka Pharmaceutical Co. Ltd., Chugai Pharmaceutical Co. Ltd., and AstraZeneca K.K. Noboru Hattori reports personal fees from AstraZeneca K.K., Chugai Pharmaceutical Co. Ltd., and Bristol Myers Squibb, and reports grants from Ono Pharmaceutical Co. Ltd., Chugai Pharmaceutical Co. Ltd., and Shino‐test Corporation. The other authors declare no conflicts of interest.

## Supporting information


**Figure S1:** Flowchart of patient enrollment.
**Figure S2:** Baseline neutrophil‐to‐lymphocyte ratio (NLR) in patients with mild checkpoint inhibitor pneumonitis (CIP) and without CIP.
**Figure S3:** The association between baseline neutrophil‐to‐lymphocyte ratio (NLR) and severe checkpoint inhibitor pneumonitis (CIP) in the pooled cohort.
**Figure S4:** Comparison of the incidence of severe checkpoint inhibitor pneumonitis (CIP) according to treatment regimen and baseline neutrophil‐to‐lymphocyte ratio (NLR) in the pooled cohort.
**Figure S5:** Comparison of receiver operating characteristic (ROC) curves for predictive performance of blood biomarkers for severe checkpoint inhibitor pneumonitis (CIP) in the validation and pooled cohorts.
**Figure S6:** Comparison of baseline neutrophil‐to‐lymphocyte ratio (NLR), NLR at the time of checkpoint inhibitor pneumonitis (CIP) diagnosis, and their absolute change according to CIP severity in the pooled cohort.
**Table S1:** Baseline characteristics according to baseline neutrophil‐to‐lymphocyte ratio (NLR) levels.
**Table S2:** Logistic regression analyses for identifying the risk of severe checkpoint inhibitor pneumonitis (CIP) in the discovery and validation cohorts.

## Data Availability

The data that support the findings of this study are available from the corresponding author upon reasonable request.
