# Supplementary material for: Baseline Neutrophil‐To‐Lymphocyte Ratio as a Risk Stratification Marker for Severe Checkpoint Inhibitor Pneumonitis in Patients With Non‐Small Cell Lung Cancer: A Two‐Cohort Study With Retrospective Discovery and Prospective Validation
Source: Respirology. 2026 Jun 4;31(9):922–32. doi: 10.1002/resp.70267 (PMC13535713; doi:10.1002/resp.70267)
Supplement: Supplementary file 1 [file RESP-31-922-s001.docx]

**Supporting Information**

**Baseline neutrophil-to-lymphocyte ratio as a risk stratification marker for severe checkpoint inhibitor pneumonitis in patients with non-small cell lung cancer: a two-cohort study with retrospective discovery and prospective validation**

Tetsu Hirakawa, Kakuhiro Yamaguchi, Shun Takao, Nobuhiro Kanaji, Naoko Matsumoto, Kozue Miyazaki, Tamio Okimoto, Chihiro Ando, Hisao Higo, Masaaki Yanai, Koji Kurose, Yasunari Nagasaki, Masako Watanabe, Kosuke Oyama, Yuko Toyoda, Yoshihiro Taguchi, Shimpei Tada, Kiyofumi Shimoji, Shinjiro Sakamoto, Yasushi Horimasu, Takeshi Masuda, Taku Nakashima, Hiroshi Iwamoto, Hironobu Hamada, Noboru Hattori

**Figure S1. Flowchart of patient enrollment**

**Figure S2. Baseline neutrophil-to-lymphocyte ratio (NLR) in patients with mild checkpoint inhibitor pneumonitis (CIP) and without CIP**

**Figure S3. The association between baseline neutrophil-to-lymphocyte ratio (NLR) and severe checkpoint inhibitor pneumonitis (CIP) in the pooled cohort**

**Figure S4. Comparison of the incidence of severe checkpoint inhibitor pneumonitis (CIP) according to treatment regimen and baseline neutrophil-to-lymphocyte ratio (NLR) in the pooled cohort**

**Figure S5. Comparison of receiver operating characteristic (ROC) curves for predictive performance of blood biomarkers for severe checkpoint inhibitor pneumonitis (CIP) in the validation and pooled cohorts**

**Figure S6. Comparison of baseline neutrophil-to-lymphocyte ratio (NLR), NLR at the time of checkpoint inhibitor pneumonitis (CIP) diagnosis, and their absolute change according to CIP severity in the pooled cohort**

**Table S1. Baseline characteristics according to baseline neutrophil-to-lymphocyte ratio (NLR) levels**

**Table S2. Logistic regression analyses for identifying the risk of severe checkpoint inhibitor pneumonitis (CIP) in the discovery and validation cohorts**

**Figure S1. Flowchart of patient enrollment**

In the discovery cohort, 102 patients with NSCLC who received anti-PD-1/PD-L1 antibody monotherapy were retrospectively analyzed. In the validation cohort, 191 patients with NSCLC who received anti-PD-1/PD-L1 antibody or in combination with anti-cytotoxic T-lymphocyte antigen-4 (CTLA-4) antibody and/or platinum-based chemotherapy as first-line therapy were prospectively analyzed. Pooled analysis was conducted in 293 patients with NSCLC.

Abbreviations: CTLA-4, cytotoxic T-lymphocyte antigen-4; ICI, immune checkpoint inhibitor; NSCLC, non-small cell lung cancer; PD-1, programmed cell death 1; PD-L1, programmed cell death ligand 1.

**Figure S2. Baseline neutrophil-to-lymphocyte ratio (NLR) in patients with mild checkpoint inhibitor pneumonitis (CIP) and without CIP**

Baseline NLR did not differ significantly between patients who developed mild CIP and those who did not developed CIP in each cohort: (a) in the discovery cohort, 6.35 [2.59–9.25] vs. 3.63 [2.62–6.28], *p* = 0.359; (b) in the validation cohort, 3.92 [2.54–5.49] vs. 3.67 [2.73–5.70], *p* = 0.653. The incidence of mild CIP did not differ significantly between patients with a higher baseline NLR (≥ 4.75) and those with a lower baseline NLR (< 4.75) in either (c) the discovery cohort (8.9% vs. 3.3%, *p* = 0.246) or (d) the validation cohort (4.9% vs. 5.0%, *p* = 0.971).

Boxes represent the interquartile range (25th–75th percentiles), solid lines within the boxes indicate the medians, and whiskers denote the 10th and 90th percentiles. Outliers were plotted as individual points. ^*^*p* < 0.05, ^**^*p* < 0.01, and ^***^*p* < 0.001, Mann-Whitney U test and Pearson’s chi-square test.

Abbreviations: CIP, checkpoint inhibitor pneumonitis; NLR, neutrophil-to-lymphocyte ratio.

**Figure S3. The association between baseline neutrophil-to-lymphocyte ratio (NLR) and severe checkpoint inhibitor pneumonitis (CIP) in the pooled cohort**

(a) The Baseline NLR was significantly higher in patients with severe CIP than in those without severe CIP (6.16 [4.15–17.41] vs. 3.67 [2.69–5.90], *p* < 0.001). (b) The incidence of severe CIP was significantly higher in patients with a higher baseline NLR (≥ 4.75) than in those with a lower baseline NLR (< 4.75) (12.0% vs. 2.7%, *p* = 0.001).

Boxes represent the interquartile range (25th–75th percentiles), solid lines within the boxes indicate the medians, and whiskers denote the 10th and 90th percentiles. Outliers were plotted as individual points. ^*^*p* < 0.05, ^**^*p* < 0.01, and ^***^*p* < 0.001, Mann-Whitney U test and Pearson’s chi-square test.

Abbreviations: CIP, checkpoint inhibitor pneumonitis; NLR, neutrophil-to-lymphocyte ratio.

**Figure S4. Comparison of the incidence of severe checkpoint inhibitor pneumonitis (CIP) according to treatment regimen and baseline neutrophil-to-lymphocyte ratio (NLR) in the pooled cohort**

The incidence of severe CIP was compared between patients with a higher baseline NLR (≥ 4.75) and those with a lower baseline NLR (< 4.75) according to treatment subgroups: (a) treatment with pembrolizumab, (b) treatment with nivolumab, (c) treatment with atezolizumab, (d) treatment with nivolumab plus ipilimumab, and (e) treatment with durvalumab plus tremelimumab.

Abbreviations: CIP, checkpoint inhibitor pneumonitis; NLR, neutrophil-to-lymphocyte ratio.

**Figure S5. Comparison of receiver operating characteristic (ROC) curves for predictive performance of blood biomarkers for severe checkpoint inhibitor pneumonitis (CIP) in the validation and pooled cohorts**

Receiver operating characteristic (ROC) curve analysis was performed to compare the predictive performance of each baseline biomarker for severe CIP in the (a) validation cohort and (b) pooled cohort. The area under the curve (AUC) was calculated for each biomarker in each cohort. Among the evaluated biomarkers, NLR showed the highest AUC for CIP in both the cohorts.

Abbreviations: AUC, area under the curve; WBC, white blood cell count; ANC, absolute neutrophil count; ALC, absolute lymphocyte count; CI, confidence interval; CIP, checkpoint inhibitor pneumonitis; NLR, neutrophil-to-lymphocyte ratio.

**Figure S6. Comparison of baseline neutrophil-to-lymphocyte ratio (NLR), NLR at the time of checkpoint inhibitor pneumonitis (CIP) diagnosis, and their absolute change according to CIP severity in the pooled cohort**

(a, b) Both baseline NLR and NLR at the time of CIP diagnosis were significantly higher in patients with severe CIP than in those with mild CIP (6.16 [4.15–17.41] vs. 4.15 [2.62–6.11], *p* = 0.040, and 7.28 [4.28–12.0] vs. 3.75 [1.82–6.83], *p* = 0.023, respectively). (c) The absolute change between baseline NLR and NLR at the time of CIP diagnosis did not differ significantly between patients with severe CIP and those with mild CIP (0.65 [-2.98–5.15] vs. -0.08 [-1.59–1.31], *p* = 0.676).

Boxes represent the interquartile range (25th–75th percentiles), solid lines within the boxes indicate the medians, and whiskers denote the 10th and 90th percentiles. Outliers were plotted as individual points. ^*^*p* < 0.05, ^**^*p* < 0.01 and ^***^*p* < 0.001, Mann-Whitney U test.

Abbreviations: CIP, checkpoint inhibitor pneumonitis; NLR, neutrophil-to-lymphocyte ratio.

**Table S1. Baseline characteristics according to baseline neutrophil-to-lymphocyte ratio (NLR) levels**

|  | Discovery cohort | | |  | Validation cohort | | |
| --- | --- | --- | --- | --- | --- | --- | --- |
|  | NLR ≥ 4.75  (n = 40) | NLR < 4.75  (n = 62) | *p*-value |  | NLR ≥ 4.75  (n = 68) | NLR < 4.75  (n = 123) | *p*-value |
| Age (years) | 73 (65–77) | 71 (65–77) | 0.853 |  | 73 (68–78) | 71 (64–76) | 0.155 |
| Sex, Male, n (%) | 28 (70.0) | 43 (69.4) | 0.945 |  | 51 (75.0) | 108 (87.8) | 0.023^*^ |
| Smoking history, Current or Former, n (%) | 32 (80.0) | 52 (83.9) | 0.617 |  | 58 (85.3) | 112 (91.1) | 0.223 |
| PS, ≥ 2, n (%) | 9 (22.5) | 13 (21.0) | 0.854 |  | 11 (16.2) | 5 (4.1) | 0.004^**^ |
| Interstitial lung disease, +, n (%) | 5 (12.5) | 13 (21.0) | 0.273 |  | 0 (0.0) | 0 (0.0) | – |
| COPD, +, n (%) | 14 (35.0) | 19 (30.6) | 0.646 |  | 20 (29.4) | 45 (36.6) | 0.316 |
| Previous thoracic RT, +, n (%) | 21 (52.5) | 13 (21.0) | 0.001^**^ |  | 3 (4.4) | 5 (4.1) | 0.909 |
| Stage, n (%) |  |  | 0.015^*^ |  |  |  | 0.054 |
| III | 0 (0.0) | 8 (12.9) |  |  | 4 (5.9) | 11 (8.9) |  |
| IV | 24 (60.0) | 41 (66.1) |  |  | 60 (88.2) | 91 (74.0) |  |
| Recurrence | 16 (40.0) | 13 (21.0) |  |  | 4 (5.9) | 21 (17.1) |  |
| Histological type, n (%) |  |  | 0.004^*^ |  |  |  | 0.219 |
| Squamous | 12 (30.0) | 5 (8.1) |  |  | 21 (30.9) | 28 (22.8) |  |
| Non-Squamous | 28 (70.0) | 57 (91.9) |  |  | 47 (69.1) | 95 (77.2) |  |
| Driver mutation, +, n (%) | 6 (15.0) | 13 (21.0) | 0.450 |  | 6 (8.8) | 7 (5.7) | 0.411 |
| PD-L1 TPS, n (%) |  |  | 0.403 |  |  |  | 0.062 |
| ≥50% | 18 (45.0) | 24 (38.7) |  |  | 34 (50.0) | 53 (43.1) |  |
| 1–49% | 10 (25.0) | 10 (16.1) |  |  | 24 (35.3) | 36 (29.3) |  |
| <1% | 2 (5.0) | 7 (11.3) |  |  | 6 (8.8) | 30 (24.4) |  |
| unknown | 10 (25.0) | 21 (33.9) |  |  | 4 (5.9) | 4 (3.3) |  |
| ICI treatment line, n (%) |  |  | 0.646 |  |  |  | – |
| 1^st^ | 14 (35.0) | 19 (30.6) |  |  | 68 (100.0) | 123 (100.0) |  |
| 2^nd^ or later | 26 (65.0) | 43 (69.4) |  |  | 0 (0.0) | 0 (0.0) |  |
| Treatment regimen, n (%) |  |  |  |  |  |  |  |
| Anti-PD-1 antibody | 37 (92.5) | 48 (77.4) | 0.046^*^ |  | 61 (89.7) | 102 (82.9) | 0.205 |
| Anti-PD-L1 antibody | 3 (7.5) | 14 (22.6) | 0.046^*^ |  | 7 (10.3) | 21 (17.1) | 0.205 |
| With anti-CTLA-4 antibody | 0 (0.0) | 0 (0.0) | – |  | 13 (19.1) | 28 (22.8) | 0.557 |
| With chemotherapy | 0 (0.0) | 0 (0.0) | – |  | 44 (64.7) | 86 (69.9) | 0.459 |

Data are presented as median (interquartile range) unless stated otherwise.

^*^*p* < 0.05 and ^**^*p* < 0.01, comparison between NLR ≥ 4.75 and NLR < 4.75 using the Mann–Whitney U test or Pearson’s chi-square test.

Abbreviations: COPD, chronic obstructive pulmonary disease; ICI, immune checkpoint inhibitor; NLR, neutrophil-to-lymphocyte ratio; PD-1, programmed cell death 1; PD-L1, programmed cell death ligand 1; PS, performance status; RT, radiotherapy; TPS, tumor proportion score.

**Table S2. Logistic regression analyses for identifying the risk of severe checkpoint inhibitor pneumonitis (CIP) in the discovery and validation cohorts**

|  | Discovery cohort (*n* = 102) | | |  | Validation cohort (*n* = 191) | | |
| --- | --- | --- | --- | --- | --- | --- | --- |
| Variables | OR | 95% CI | *p*-value |  | OR | 95% CI | *p*-value |
| Univariate analysis |  |  |  |  |  |  |  |
| Age, years | 0.984 | 0.916–1.071 | 0.681 |  | 1.003 | 0.947–1.073 | 0.936 |
| Sex, male | 2.769 | 0.446–53.494 | 0.305 |  | Not available^†^ | Not available^†^ | 0.041^*^ |
| Smoking history, Current or Former | Not available^†^ | Not available^†^ | 0.093 |  | Not available^†^ | Not available^†^ | 0.104 |
| PS, ≥ 2 | 0.587 | 0.030–3.708 | 0.612 |  | Not available^†^ | Not available^†^ | 0.159 |
| Interstitial lung disease, + | 1.975 | 0.267–10.095 | 0.460 |  | – | – | – |
| History of COPD, + | 0.826 | 0.114–4.071 | 0.823 |  | 1.115 | 0.283–3.839 | 0.867 |
| Previous thoracic RT, + | 0.788 | 0.109–3.878 | 0.779 |  | 6.444 | 0.864–33.006 | 0.066 |
| Stage, Ⅳ or recurrence | Not available^†^ | Not available^†^ | 0.276 |  | Not available^†^ | Not available^†^ | 0.173 |
| Histological type, Squamous | Not available^†^ | Not available^†^ | 0.103 |  | 0.629 | 0.094–2.551 | 0.545 |
| Driver mutation, positive | 0.713 | 0.037–4.543 | 0.753 |  | Not available^†^ | Not available^†^ | 0.206 |
| PD-L1 TPS, ≥50% | 0.550 | 0.076–2.694 | 0.474 |  | 1.004 | 0.292–3.597 | 0.995 |
| ICI treatment line, 1^st^ | Not available^†^ | Not available^†^ | 0.017^*^ |  | – | – | – |
| ICI treatment, anti-PD-1 antibody | 1.215 | 0.189–23.757 | 0.858 |  | Not available^†^ | Not available^†^ | 0.058 |
| ICI treatment, with anti-CTLA-4 antibody | – | – | – |  | 1.401 | 0.296–5.111 | 0.638 |
| ICI treatment, with chemotherapy | – | – | – |  | 1.268 | 0.352–5.947 | 0.729 |
| NLR, ≥ 4.75 | 10.765 | 1.741–207.705 | 0.008^**^ |  | 3.414 | 0.992–13.453 | 0.052 |
| Alb, ≥ 3.5 g/dL | 0.620 | 0.117–2.958 | 0.544 |  | 0.357 | 0.099–1.234 | 0.102 |
| CRP, ≥ 1.0 mg/dL | 0.704 | 0.133–3.357 | 0.656 |  | 2.395 | 0.698–9.411 | 0.166 |
|  |  |  |  |  |  |  |  |
| Multivariate analysis |  |  |  |  |  |  |  |
| Smoking history, Current or Former | Not available^†^ | Not available^†^ | 0.070 |  | Not available^†^ | Not available^†^ | 0.077 |
| NLR, ≥ 4.75 | 11.769 | 1.877–228.414 | 0.006^**^ |  | 3.706 | 1.070–14.680 | 0.039^*^ |

^†^OR (odds ratio) and 95% confidence interval (CI) could not be calculated because only one group developed severe CIP.

^*^p < 0.05, ^**^p < 0.01, and ^***^p < 0.001, logistic regression analysis.

Abbreviations: Alb, albumin; CI, confidence interval; CIP, checkpoint inhibitor pneumonitis; COPD, chronic obstructive pulmonary disease; CRP, C-reactive protein; CTLA4, cytotoxic T-lymphocyte antigen 4; ICI, immune checkpoint inhibitor; OR, odds ratio; PD-1, programmed cell death-1; PD-L1, programmed cell death ligand-1; PS, performance status; TPS, tumor proportion score.
